# A voltage and current measurement dataset for plug load appliance identification in households

**DOI:** 10.1038/s41597-020-0389-7

**Published:** 2020-02-12

**Authors:** Roberto Medico, Leen De Baets, Jingkun Gao, Suman Giri, Emre Kara, Tom Dhaene, Chris Develder, Mario Bergés, Dirk Deschrijver

**Affiliations:** 1 Department of Information Technology, Ghent University - imec, Technologiepark-Zwijnaarde 126, 9052 Ghent, Belgium; 2 Civil & Environmental Engineering, Carnegie Mellon University, Pittsburgh, PA 15213-3890 United States

**Keywords:** Energy supply and demand, Energy and behaviour, Energy efficiency

## Abstract

This paper presents the Plug-Load Appliance Identification Dataset (PLAID), a labelled dataset containing records of the electrical voltage and current of domestic electrical appliances obtained at a high sampling frequency (30 kHz). The dataset contains 1876 records of individually-metered appliances from 17 different appliance types (e.g., refrigerators, microwave ovens, etc.) comprising 330 different makes and models, and collected at 65 different locations in Pittsburgh, Pennsylvania (USA). Additionally, PLAID contains 1314 records of the combined operation of 13 of these appliance types (i.e., measurements obtained when multiple appliances were active simultaneously). Identifying electrical appliances based on electrical measurements is of importance in demand-side management applications for the electrical power grid including automated load control, load scheduling and non-intrusive load monitoring. This paper provides a systematic description of the measurement setup and dataset so that it can be used to develop and benchmark new methods in these and other applications, and so that extensions to it can be developed and incorporated in a consistent manner.

## Abstract (summary)

**Table:** 

Measurement(s)	electrical current • Voltage
Technology Type(s)	current clamp • oscilloscope
Factor Type(s)	location • type of electrical appliance
Sample Characteristic - Location	Commonwealth of Pennsylvania

Machine-accessible metadata file describing the reported data: 10.6084/m9.figshare.11733594

## Background & Summary

The Plug-Load Appliance Identification Dataset (PLAID) is a public dataset consisting of voltage and current measurements from different electrical household appliances sampled at 30 kHz. All appliances are monitored *individually*: they are submetered and the data traces captured over a few seconds include the activation of the appliances. Additionally, some of them are also monitored when active *simultaneously*: their aggregated consumption is measured and the data captured over a few minutes contains the activation and deactivation of a subset of the appliances. Activations and deactivations are characterized by events in the current and voltage signals.

In total, 17 different appliance types (e.g., refrigerators, microwave ovens, etc.) are measured in 65 different locations for the submetered data, and 13 different appliance types (a subset from those used for the submetered data) are measured at one single location for the aggregated data. Not all appliance types are available in all different locations. In total, the dataset contains 330 different appliances (i.e., different appliance models for each of the 17 different appliance types). For some appliances (approximately 10% of them), multiple operating modes were monitored.

The dataset has grown over the years^1,2^: published in 2014 and^3,4^ published in 2017 contain, respectively, 55% and 38% of the currently available submetered data. Note that some of the original measurements from^2^ and^4^ have been removed in this version. Specifically, measurements were removed if the following conditions on voltage (*V*) and current (*I*) were not met in steady state:

$$\begin{array}{lll}110\,V &  < = & {V}_{RMS} < \,=130\,V\\ {\rm{\max }}\,I &  < = & 20\,A\end{array}$$

The main contributions of this paper are that it:

- Compiles all previous PLAID dataset releases into a single reference dataset;
- Augments the available submetered data with additional 7% of data;
- Adds aggregated data measurements;
- Provides all data and metadata into a unified and structured format for a more convenient usage.

Moreover, the added appliances and location are different as compared to previous versions, and include new appliance types. Our goal is to continuously expand this dataset by incorporating additional measurements of appliances at different locations. To facilitate this goal, this paper describes the technical procedure to consistently replicate the setup. The aim of this paper is thus to advocate and streamline the usage and potential extension of PLAID as a publicly available resource for NILM research for both high-frequency submetered and aggregated data. The data described in this paper is accessible at^5^.

PLAID can be used in two ways. First, the high resolution submetered appliance measurements (30 kHz) can be used to automate the labelling of submetered data, enabling the possibility for appliance classification (i.e., being able to classify appliance types from just voltage and current measurements). This knowledge is interesting for smart plugs^6^ that are used for smart grid and building-level energy management applications such as automated load control^7^ and load scheduling^8^. In addition to appliance classification, this data can also be used to create an appliance power consumption inventory. As the submetered data is captured in different houses, the generalization of the labelling methods across houses can be tested. Second, the high resolution aggregated appliance measurements (30 kHz) can be used to learn how to dissagregate the total current consumption measured at the main feed of a household at high frequency. This is known as non-intrusive load monitoring (NILM)^9^. Two important steps in NILM are event detection^10^ and load identification^11^. This dataset provides the means to learn and implement both tasks on high frequency data. The obtained information can also be used to identify energy consumption and to monitor the deterioration of appliances.

Table 1 shows similar datasets that are publicly available. PLAID is distinct because it contains submetered and aggregated data sampled at a frequency higher than 1 Hz. Only two other datasets (WHITED^12^ and COOLL^13^) contain submetered data sampled at a frequency higher than 1 Hz. All the others, like ACS-F2^14^ and Tracebase^15^, contain submetered data sampled at a frequency lower than 1 Hz. From these last datasets, only two, i.e., REDD^16^, and UK-DALE^17^, contain aggregated data sampled at a frequency higher than 1 Hz. HELD1^18^ contains aggregated measurements at a frequency of 4 kHz, where up to ten devices can be switched on/off simultaneously. All the other datasets, i.e. DRED^19^, Dataport^20^, REFIT^21^ and AMPds2^22^ contain aggregated data sampled at a frequency lower than 1 Hz.

**Table 1:** An overview of PLAID and similar datasets in terms of submetered data sampled at a frequency <1 Hz or ≥1 Hz, aggregated data sampled at a frequency <1 Hz or ≥1 Hz, different appliance operating modes and the number of different buildings.

	Sampling Frequency				Appliance operating modes	# of buildings
	Submetered		Aggregated			
	<1 Hz	≥1 Hz	<1 Hz	≥1 Hz		
PLAID		✓		✓	multiple	65
WHITED^12^		✓			on, off	
COOLL^13^		✓			on, off	
ACS-F2^14^	✓				on, off	
Tracebase^15^	✓				on, off	
REDD^16^	✓			✓	on, off	2
UK-DALE^17^	✓			✓	on, off	6
DRED^19^	✓		✓		on, off	
Dataport^20^	✓		✓		on, off	1200+
REFIT^21^	✓		✓		on, off	20
AMPds2^22^	✓		✓		on, off	
HELD1^18^				✓	on, off	

## Methods

First, the hardware used to monitor the appliances is described. Next, we describe the selected appliances and their occurrence in the different households. The next two subsections explain how the appliances are submetered and aggregated. Finally, known issues and details on the data and code availability are given in the last subsections.

### Monitoring set-up

**Fig. 1:**
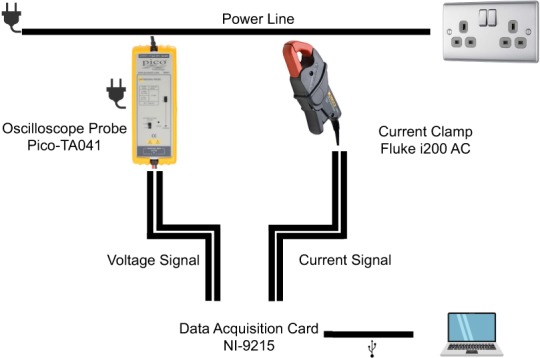
The measurement set-up for capturing the data.

All electrical measurements were collected using a National Instruments (NI-9215) data acquisition card (https://www.ni.com/data-acquisition/). The NI-9215 includes four simultaneously sampled analog input channels paired with a 16-bit analog-to-digital converter (ADC) that we use to collect voltage and current measurements. These are stored in a computer via a USB connection, as shown in Fig. 1.

To measure the different appliances, these were connected to the power strip. This power strip has a negligible amount of power consumption as a small lamp was burning indicating the activity of the power strip. As a consequence, this small load is measured during the data collection. From this power strip, the current and voltage are measured.

Current is measured with a Fluke i200 AC current clamp (https://en-us.fluke.com/products/all-accessories/Fluke-i200s.html) that has a cut-off frequency of 10 kHz, allowing us to sample signals with frequency content up to 5 kHz according to the Nyquist-Shannon sampling theorem^23^. These current clamps have a measuring range of 0.5 to 240 A, with less than 3.5% + 0.5 A accuracy in the 48–65 Hz range, and less than 6° phase shift for the amplitudes of interest in this study. It is important to note that if the current is sampled at a high frequency, it is necessary to have a clamp with a high cut-off frequency. Some of the existing datasets with high sampling frequency did not account for this (e.g., BLUED^24^ used a current transformer with a cut-off frequency of ~300 Hz). The Fluke i200 is connected to the NI-9215, see Fig. 1.

Voltage is measured with a Pico-TA041 Oscilloscope probe (https://www.picotech.com/accessories/high-voltage-active-differential-probes/25-mhz-700-v-differential-probe). The TA041 is an active differential probe suitable for high common-mode voltage measurement applications up to ±700 V (DC + peak AC). It can be used with signal frequencies of up to 25 MHz. Because the active probes significantly reduce capacitive loading, they are able to achieve fast signal measurements with much better signal fidelity making them well suited for high frequency measurements. As with the current clamp, the Pico-TA041 is connected to the NI-9215, see Fig. 1.

The NI-9215 converts the analog voltage and current signals into digital signals and sends them via an USB-connection to a computer. The digital signals have an effective resolution of approximately 0.03 A for current, and 0.03 V for voltage. Libraries for different programming languages (e.g., Python, C++, MATLAB, and LabVIEW) can be used to communicate with the NI-9215 under the condition that the correct drivers are installed. We used MATLAB and LabVIEW and stored the data in comma-separated values (CSV) files. Reference scripts for replicating this process are also made available as part of the dataset.

Though the specific hardware used in our instrumentation setup can be costly, low-cost alternatives with similar or better specifications have become available in recent years (e.g.^25–32^).

### Selected homes and appliances

In total, 17 appliance types were measured at 65 locations. These include one lab environment and 64 households. These households were recruited via an email campaign and mainly consist of graduate student homes. All the households are located in Pittsburgh, Pennsylvania, USA.

Table 2 gives an overview of the 17 appliance types, their occurrence in the 65 locations (number of appliances) and the number of times these were monitored/activated (number of instances), both for the submetered and aggregated case. For example, for the refrigerator appliance type, 28 physically different refrigerators are monitored separately multiple times, leading to 100 instances of this appliance type. One of these refrigerators is monitored 79 times when other appliances were active or were turned on. For six appliances types that were located in the lab environment, only one appliance is monitored. Those appliances were also used to generate the aggregate measurements. Note, that there is less data of the blender appliance type compared to the other appliance types, as it broke down in the middle of the experiment.

**Table 2:** Summary of the different appliances in PLAID. R = resistive, I = inductive, NL = non-linear.

Appliance		Submetered		Operating modes	Aggregated	
type	load	# of appl.	# of inst.		# of appl.	# of inst.
Air Conditioner	NL	27	204	[high cool, high fan, low cool, low fan]	1	160
Blender	I	1	2	[off, on]	1	51
Coffeemaker	R	1	10	[off, on]	1	106
Compact Fluorescent Light	NL	45	230	[off, on]	1	104
Fan	I	31	220	[high, medium, low]	1	102
Refrigerator	I	28	108	[off, on, unknown]	1	167
Hairdryer	R	36	246	[high warm, low warm, high hot, low hot]	0	0
Hair iron	NL	1	10	[off, on]	1	98
Heater	R	15	85	[high, low]	0	0
Incandescent Light Bulb	R	33	157	[off, on]	1	11
Laptop	NL	46	216	[off, on]	1	90
Microw. oven	NL	32	200	[high, medium]	0	0
Soldering iron	NL	1	20	[off, on]	1	218
Vacuum cleaner	I	15	83	[off, on]	1	98
Washing Machine	NL	16	75	[off, on]	0	0
Water kettle	R	1	10	[off, on]	1	109
Total		1876			1314	

All the appliances were activated by connecting them to the power strip and turning on the switch if present. However, the following remarks need to be given concerning activation assumptions:

- The blender was kept empty during the experiments.
- The refrigerator was activated after it warmed up by opening the door. This ensured the motor would activate.
- An unknown mode of the refrigerator was activated by plugging in the refrigerator twice shortly after each other. The second time, the unknown mode is activated.
- The soldering iron has a two-phase activation process: around 6 seconds after activation, there is an increase in power consumption. The two events are stored in two separate files, both with the label ‘soldering iron’.

### Submetered appliances

Each time an appliance is activated, a state transition (event) will happen^10^. When the appliances are monitored individually, i.e., submetered, the activation is measured together with some seconds of the steady state following this activation. This measurement captures the transient start-up containing information of the present electrical components and possible present inertia. The deactivation of the appliances is not measured because then the electrical circuit is disconnected and appliance specific information is no longer present. The recorded steady state duration ranges from 1 to 20 seconds.

Besides monitoring the activation of the appliances, the following meta-data is stored, when available:

- Manufacturing data of the appliance: the brand, manufacturing year, model number, appliance type (first column of Table 2), load type, and the rated current, voltage and power consumption values.
- Information concerning the data capturing process: the time of data collection expressed in month and year, the sampling frequency, the total measurement duration, and the specific operating mode that was measured.
- The location identifier, which is a string (e.g., ‘house5’ or ‘CMU lab’).

The current and voltage measurements themselves are stored in separate CSV files. The measurement is stored in two columns, one for the the current expressed in ampere and the other one for the voltage expressed in volt. The precision of the numbers is three decimals. As the sampling rate was kept constant, there was no need to associate each measurement with a timestamp. The time that has passed relative to the beginning of the file can be calculated using the sampling frequency (e.g., for a frequency of 30 kHz, the 30000^th^ point occurs one second after the start).

The meta-data is stored in one JavaScript Object Notation (json) file which contains for each measurement an attribute-value pair with the CSV file name of the measurement file, as attribute and the meta-data of the measurement in question as the value. The meta-data itself is also structured as attribute-value pairs as described in Box 1.

### Aggregated appliances

To measure the aggregated signals, several appliances are activated one after another. Different from the submetered case, the deactivation is also monitored. This is done because other appliances may still be running after deactivation. The 13 appliances that were present in the lab environment were used to create the aggregated data (see Table 2). The goal of this dataset is to capture the signal characteristics for combined operation of appliances. Full coverage of all the combinatorial possibilities would have been impractical. Indeed, there are 312 combinations of 2 appliances that can be made from 13 appliances. This amounts to $$4\cdot \left(\begin{array}{c}13\\ 2\end{array}\right)$$ combinations. The multiplication factor 4 refers to the different order in which 2 appliances can be activated and deactivated under the condition that first the 2 appliances must be activated before one can be deactivated. Activating more than two appliances each in turn, becomes intractable as the number of combinations grows exponentially with the number of appliances.

To make the amount of combinations more tractable, the following division is used: appliance types can be linear (L) or non-linear (NL) loads. A load is linear if there is a linear relationship between its current drawn and the supplied voltage. Some loads, such as these containing transistors and other electronics, do not behave in this way and are called non-linear loads. The linear loads can be resistive (R), capacitive (C) or inductive (I). Examples of a resistive, capacitive, and inductive loads are respectively a light bulb, a battery, and a motor. An example of a non-linear load is a computer. The grouping for the appliances present in the lab are given in the first column of Table 2 between brackets. As can be seen, there are no purely capacitive loads available, leaving the following groups: R, I and NL. The following combinations in and between the groups are measured:

- *Two different appliances of the same group* are selected (e.g., *A* and *B*) and combined in all possible ways under the condition that first the two appliances must be activated before one can be deactivated. All possible selections of appliances *A* and *B* for each group are measured. For example, for the resistive group consisting of 4 appliances, there are 6 different selections of two appliances *A* and *B*, and each is combined in 4 ways, leading to 24(=6 · 4) measurements.
- *Two different appliances, each of a different group*, are selected and combined in all possible ways under the condition that first the two appliances must be activated before one can be deactivated (see above). All possible selections of two different appliances, each of a different group are measured. As the resistive, inductive and non-linear group consists of 4, 5, and 4 appliances respectively, this leads to 56(=4 · 5 + 4 · 4 + 5 · 4) selections of two different appliances. As each selection is combined in four possible ways, in total there are 224(=56 · 4) measurements. Note that some of the combinations with the blender are missing because it broke down before the end of the experiments.
- *Three different appliances, each from one group*, are selected and combined in a random way under the conditions that three appliances must be all activated before one is deactivated and that the order of activation is the same as deactivation. As the number of possible appliance selections and combinations is too large to cover exhaustively, a random generator is used to select the three appliances and their order. This is repeated 60 times.

Combining the appliances in this way allows us to investigate the influence that appliances of the same or different groups have on each other. Investigation of this data will point out if further elaborating this dataset is necessary. Each of these measurements is only done once.

**Fig. 2:**
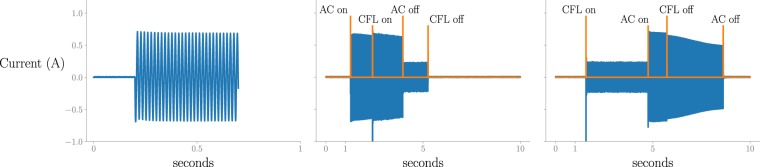
An example of the aggregated data, where appliances are (de)activated during the transient behavior of an air conditioner (AC). (**a**) The transient of the current consumption of an AC is shown (submetered/1825.csv). (**b**) The CFL is activated during the transient of the AC (aggregated/484.csv). (**c**) The CFL is deactivated in the transient behaviour of the AC (aggregated/485.csv).

A special case of aggregating appliances is when an appliance is (de)activated during the transient behavior of another appliance. In Fig. 2a, an example is given of the transient behavior of the air conditioner. When an appliance is (de)activated during the transient phase, it is seen that its behavior before/after the event is different. The AC is the only appliance in PLAID with a sufficiently large and slow transient behavior that makes it possible to simultaneously (de)activate appliances. The other appliances (except for the blender, laptop charger, refrigerator and refrigerator defroster) were either activated or deactivated at 5 different random time instances during the transient of the AC. An illustration is shown in Fig. 2b,c. In the end, 80(=8 · 5 + 8 · 5) measurements for this special case are captured. This was not done for the blender as it already broke down and not for the laptop charger, refrigerator and refrigerator defroster as these appliances are activated by connecting the plug to the power line, and it was not feasible to accomplish this within the time frame wherein the transient behavior takes place.

**Fig. 3:**
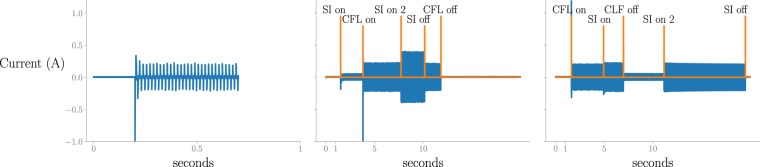
An example of the aggregated data, where appliances are (de)activated during the first step of a soldering iron (SI)’s activation. (**a**) The transient of the current consumption of the CFL is shown (submetered/1745.csv). (**b**) The CFL is activated during the first phase of activation of the soldering iron (SI) (aggregated/558.csv). (**c**) The CFL is deactivated during the first phase of activation of the soldering iron (SI) (aggregated/559.csv).

Another special case is when the soldering iron with the two-phase activation process is used (see Fig. 3a). In the previously described measurements, other appliances are only (de)activated when the soldering iron reached the second step of its activation. To complete the dataset, we also captured data where appliances are (de)activated during the first step of the soldering iron’s activation. More specifically for an appliance A two measurements are captured in the following manner:

- Appliance A is activated between the first and second step of the soldering iron’s activation. Once the activation of both appliances is complete, the soldering iron and A are deactivated each in turn, as shown in Fig. 3b.
- Appliance A and the soldering iron are activated each in turn. Then, appliance A deactivated in between the first and second step of the soldering iron’s activation, as shown in Fig. 3c.

For each appliance type, the above measurements are only done once, as repeating the experiments would result in almost identical events, since the time and the current consumption between the two activation steps is always the same. This is done for every other appliance, resulting in 24(=2 · 12) measurements.

The measurements are stored in CSV files. Table 3 gives an overview of the files corresponding to each experiment. The meta-data follows the same structure as for the submetered data and extends it by adding an array of appliances monitored in the file. Each appliance is characterized by its manufacturing data (see meta-data of submetered data), and timestamps of activation and deactivation. The timestamps are expressed using indices from which the time passed since the start of the file can be calculated using the known sampling frequency of 30 kHz. The index represents the moment the appliance is activated and not the moment the appliance reaches steady state. Note that the soldering iron induces two events when it is activated, one for each activation phase, and both are labelled. Just as for the meta-data of the submetered data, the meta-data of the aggregated data is structured as attribute-value pairs as described in Box 1, where the additions are put in italic.

**Table 3:** An overview of the correspondence between file number and experiment for the aggregated data.

Files	Experiment
1–474	2 or 3 appliances active,
	on/off outside transient,
	on/off in second activation phase of soldering iron
475–554	AC and other appliance,
	on/off during AC transient,
	on/off in second activation phase of soldering iron
555–576	Soldering iron and other appliance,
	on/off outside transient,
	on/off in first activation phase of soldering iron

### Known Issues

**Fig. 4:**
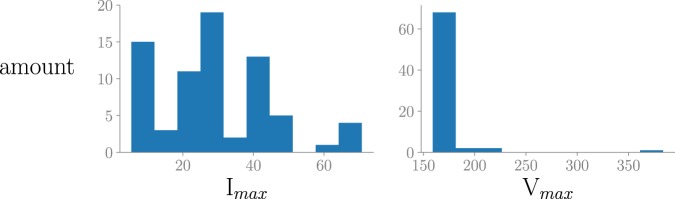
The histograms of maximal current and voltage values in steady state for the measured vacuum cleaners.

Some issues are present in PLAID. When monitoring the appliances individually in the 2014 version (the submetered files with identifiers going from 1 to 1027), the calibration was not checked every time when the set-up changed places. As an example, the histogram in Fig. 4 shows the distribution of maximal current and voltage values for the vacuum appliance type, indicating a great variation in the values as the maximal current values range from 5.4 A to 70.7 A and the maximal voltage values range from 159.02 V to 383.7 V. Some of the variance in the values can be explained by the fact that there are 15 different vacuum cleaners, but the smallest values suggest a calibration error. As a consequence, a data normalization step is needed for further processing. This must be done by the user.

Table 2 also shows that the data is very imbalanced (e.g., 85 instances for the heater appliance type compared to the 230 instances for the compact fluorescent lamp appliance type). This imbalance needs to be considered in evaluation of, e.g., automatic classification^3^.

An additional minor issue is that the meta-data concerning the manufacturing of the appliances is quite often left blank by the measurer, as can been seen in Table 4. Having this information could be valuable for comparing the power consumption between different generations of appliances or different brands.

**Table 4:** The number of instances for which the metadata fields are completed.

Meta-data	Submetered #/Total (%)		Aggregated #/Total (%)	
brand	823/1876	(43.87%)	1254/1305	(96.09%)
current consumption	449/1876	(23.94%)	759/1305	(58.16%)
manufacturing year	23/1876	(1.23%)	0/1305	(0.00%)
model number	581/1876	(30.97%)	90/1305	(6.9%)
on	N/A		1305/1305	(100%)
off	N/A		1305/1305	(100%)
voltage consumption	654/1876	(34.86%)	1087/1305	(83.30%)
wattage	452/1876	(24.09%)	700/1305	(53.64%)
capturing moment	1876/1876	(100%)	576/576	(100%)
sampling frequency	1876/1876	(100%)	576/576	(100%)
total time	1876/1876	(100%)	576/576	(100%)
measured mode	1876/1876	(100%)	576/576	(100%)
location identifier	1876/1876	(100%)	576/576	(100%)
appliance type	1876/1876	(100%)	1305/1305	(100%)

Note that for the aggregated data, the total number of instances for the manufacturing meta-data is larger than for the other meta-data, this because multiple appliances can be activated at the same time.

Box 1. Format of the meta-data files for submetered data ‘appliance’: {

‘brand’: ‘’,

‘current’: ‘’,,

‘load’:‘’,

‘manufacture_year’: ‘’,

‘model_number’: ‘’,

‘notes’: ‘’,

‘type’: ‘’

‘voltage’: ‘’},

‘wattage’: ‘’},

‘header’: {

‘collection_time’: ‘’,

‘notes’: ‘’,

‘sampling_frequency’: ‘’},

‘instances’:‘length’: ‘’,

‘status’: ‘’},

‘location’: ‘’}

## Data Records

Meta-data for both submetered and aggregated data are stored in JavaScript Object Notation (.json) with the format described in Boxes 1 and 2 respectively. The number of instances for which the metadata fields are completed is shown in Table 4. The data files referenced in the meta-data are stored in CSV (.csv) format. Each.csv file is numbered, and an overview on the mapping between file number and experiment can be found in Table 3  ^5^. Moreover, the data is made available in the HDF5 format as a single hierarchical file. Here, two groups are present (‘*aggregated*’ and ‘*submetered*’): each group consists of several datasets, each corresponding to one raw data file. These are indexed with their ID and the corresponding metadatas are stored as attribute of the datasets. When using Python, the following query can be used to, e.g., retrieve the submetered file with ID 100 and its metadata, using the h5py (https://github.com/h5py/h5py) package:

f = h5py.File(‘plaid.hdf5’, $\bigsqcup$ ‘r’)

d = f[‘submetered’][‘100’]

metadata = d.attrs[‘metadata’]

Box 2. Format of the meta-data files for aggregated data ‘appliances’: [{

‘brand’: ‘’,

‘current’: ‘’,

‘load’: ‘’,

‘manufacture_year’: ‘’,

‘model_number’: ‘’,

‘notes’: ‘’,

‘*on*’: ‘’,

‘*off*’: ‘’,

‘type’: ‘’,

‘voltage’: ‘’,

‘wattage’: ‘’}, …],

‘header’: {

‘collection_time’: ‘’,

‘notes’: ‘’,

‘sampling_frequency’: ‘’},

‘instances’: {

‘length’: ‘’,

‘status’: ‘’},

‘location’: ‘’}

## Technical Validation

PLAID can be used for different use cases that involve appliance recognition from electrical data. An advantage of this dataset is that the same appliance type is measured in different houses. In this section, we check if different appliances of the same type have a similar power profile, using the submetered data that was correctly calibrated. This can give insight whether or not it is justified to combine data from different brands within the same appliance type.

**Fig. 5:**
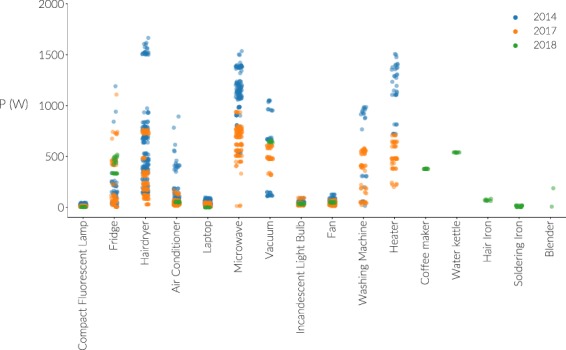
The power draw (W) of the appliances present in the dataset, across the different versions. Per appliance type, the power consumption of each measurement is shown as a dot, whose color indicates the source dataset version.

**Fig. 6:**
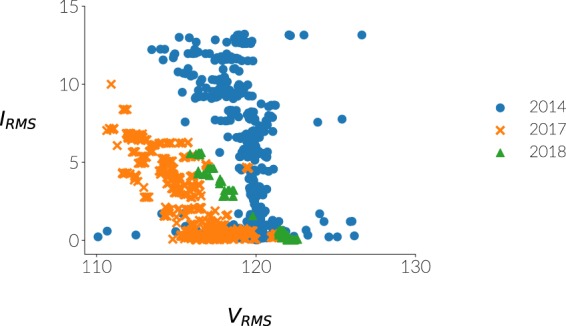
A scatterplot of the RMS values for voltage *V*_*RMS*_ and current *I*_*RMS*_ across the different versions of the dataset.

In Fig. 5, the active power consumption for the appliance types is shown. The active power for one cycle is calculated from the current and voltage signal in the following manner:

1 $$P=\frac{1}{n}\mathop{\sum }\limits_{i=1}^{n}{I}_{i}{V}_{i}$$

where *n* is the total number of samples in a cycle, *I*_i_ and *V*_i_ are respectively the *i*th sample of current and voltage of a steady state cycle of respectively the current and voltage. Figure 5 shows that the power draw of same type appliances between different brands can vary significantly. For example, the power consumption of the microwave oven varies from an average of around 500 W for the 2017 version to around 1250 W for the 2014 version. In Fig. 6, the RMS values for voltage and current of all measurements are shown, showing differences between the various versions of the dataset. This implies that the appliance recognition generalization on different houses will not be straightforward using power- or RMS-related features only, and others must be examined as well. More details on the distinguishability of individual appliances using the PLAID data can be found in^3^, where the submetered data from^4^ is used.

## Usage notes

The PLAID data is provided in CSV files and can be extracted using the common programming languages and software packages (e.g., Python, MATLAB). The dataset concerning the submetered data has grown over time. The data in^2^ corresponds to file identifiers 1–1027, while measurements from^4^ use the identifiers 1028–1745. For this paper, additional submetered data is captured, which is stored in files 1745–1877. Note that only for the files 1028–1745, multiple operating modes are considered (not only binary on/off). This versioning can also be easily achieved when the data is accessed. The current version of PLAID is available on *figshare* (figshare.com)^5^. If the dataset keeps growing in the future, *figshare* will enable controlled growth of the dataset, since versioning is available. On the repository, the following files can be found:

- *metadata*_*submetered*.*json*: the metadata for all submetered data in JSON format;
- *metadata*_*aggregated*.*json*: the metadata for all aggregated data in JSON format;
- *submetered*.*zip*: an archive containing the submetered data in CSV format;
- *aggregated*.*zip*: an archive containing the aggregated data in CSV format;
- *plaid*_*hdf5*.*zip*: an archive containing all the data in HDF5 format;
- *collecting*_*data*.*m*, *collecting*_*data*.*vi*: MATLAB and LabVIEW scripts used for capturing the data.

## Data Availability

The complete PLAID dataset and all mentioned scripts are available in^5^. In the same repository, code written to capture the data can be found. The files are two scripts, namely ‘collecting_data.vi’ (written with LabVIEW) and ‘collecting_data.m’ (written in MATLAB).

## References

[1] Gao, J., Giri, S., Kara, E. C. & Bergés, M. PLAID: a public dataset of high-resoultion electrical appliance measurements for load identification research: demo abstract. *Proc. acm conference on embedded systems for energy-efficient buildings*, 198–199 (2014).

[2] Gao J, Giri S, Kara EC, Bergés M (2020). figshare.

[3] De Baets, L. *et al*. Handling imbalance in an extended plaid, Sustainable Internet and ICT for Sustainability (SustainIT). *IEEE* (2017).

[4] De Baets L (2020). figshare.

[5] Medico R (2020). figshare.

[6] Hicham, E., Helal, A., Abdulrazak, B. & Jansen, E. Self-sensing spaces: smart plugs for smart environments. *Proceedings of the 3rd International Conference on Smart Homes and Health Telematics*, 91–98 (2005).

[7] Mohsenian-Rad A-H, Alberto L-G (2010). Optimal residential load control with price prediction in real-time electricity pricing environments. IEEE transactions on Smart Grid.

[8] Du P, Ning L (2011). Appliance commitment for household load scheduling. IEEE transactions on Smart Grid.

[9] Abubakar I, Khalid SN, Mustafa MW, Shareef H, Mustapha M (2017). Application of load monitoring in appliances’ energy management–a review. Renewable and Sustainable Energy Reviews.

[10] De Baets L, Develder C, Dhaene T, Deschrijver D (2017). On the Bayesian optimization and robustness of event detection methods in nilm. Energy and Buildings.

[11] De Baets L, Ruyssinck J, Develder C, Dhaene T, Deschrijver D (2018). Appliance classification using vi trajectories and convolutional neural networks. Energy and Buildings.

[12] Kahl, M., Haq, A. U., Kriechbaumer, T. & Jacobsen, H.-A. Whited-a worldwide household and industry transient energy data set. *Proc. 3rd international workshop on non-intrusive load monitoring* (2016).

[13] Picon, T. *et al*. COOLL: Controlled On/Off Loads Library, a Public Dataset of High-Sampled Electrical Signals for Appliance Identification, Preprint at, https://arxiv.org/abs/1611.05803 (2016).

[14] Ridi, A., Gisler, C. & Hennebert, J. ACS-F2 – A new database of appliance consumption signatures. *6th International Conference of Soft Computing and Pattern Recognition (SoCPaR)*, 145–150 (2014).

[15] Reinhardt, A. *et al*. On the accuracy of appliance identification based on distributed load metering data. *Sustainable Internet and ICT for Sustainability (SustainIT)*, 1–9 (2012).

[16] Kolter, J. Z. & Johnson, M. J. REDD: A public data set for energy disaggregation research. *Workshop on Data Mining Applications in Sustainability (SIGKDD)*, 59–62 (2011).

[17] Kelly, J. & Knottenbelt, W. The UK-DALE dataset, domestic appliance-level electricity demand and whole-house demand from five UK homes. *Scientific data*, **2** (2015). 10.1038/sdata.2015.7 PMC4432654 25984347

[18] Held, P., Mauch, S., Saleh, A., Benyoucef, D. & Abdeslam, D. O. Home equipment laboratory dataset for non-intrusive load monitoring. *SIGNAL 2018 Editors* (2018).

[19] Nambi, A. S. U., Lua, A. R. & Prasad, V. R. Loced: Location-aware energy disaggregation framework. *Proceedings of the 2nd acm international conference on embedded systems for energy-efficient built environments*, 45–54 (2015).

[20] Parson, O. *et al*. Dataport and NILMTK: A building data set designed for non-intrusive load monitoring. *IEEE Global Conference on Signal and Information Processing (GlobalSIP)*, 210–214 (2015).

[21] Murray, D., Stankovic, L. & Stankovic, V. An electrical load measurements dataset of united kingdom households from a two-year longitudinal study. *Scientific data*, **4** (2017). 10.1038/sdata.2016.122 PMC5315495 28055033

[22] Makonin, S., Ellert, B., Bajić, I. V. & Popowich, F. Electricity, water, and natural gas consumption of a residential house in Canada from 2012 to 2014. *Scientific data*, **3** (2016). 10.1038/sdata.2016.37 PMC4896126 27271937

[23] Shannon EC (1949). Communication in the presence of noise. Proceedings of the IRE.

[24] Anderson, K. *et al*. Blued: A fully labeled public dataset for event-based non-intrusive load monitoring research. *Proceedings of the 2nd Workshop on Data Mining Applications in Sustainability* (2011).

[25] Jiang, X., Dawson-Haggerty, S., Dutta, P. & Culler, D. Design and implementation of a high-fidelity ac metering network. book *International conference on information processing in sensor networks*, 253–264 (2009).

[26] Jadhav, A. R. & Rajalakshmi, P. Iot enabled smart and secure power monitor. *IEEE region 10 symposium (tensymp)*, 1–4 (2017).

[27] Klemenjak C, Egarter D, Elmenreich W (2016). Yomo: the arduino-based smart metering board. Computer Science-Research and Development.

[28] Klemenjak, C., Jost, S. & Elmenreich, W. Yomopie: A user-oriented energy monitor to enhance energy efficiency in households. *IEEE conference on technologies for sustainability (sustech)*, 1–7 (2018).

[29] Kriechbaumer, T., Ul Haq, A., Kahl, M. & Jacobsen, H. A. Medal: A cost-effective high-frequency energy data acquisition system for electrical appliances. *Proceedings of the eighth international conference on future energy systems*, 216–221 (2017).

[30] Makonin, S. *et al*. Inspiring energy conservation through open source metering hardware and embedded real-time load disaggregation. *IEEE PES Asia-Pacific Power and Energy Engineering Conference (APPEEC)*, 1–6 (2013).

[31] Oberloier S, Pearce JM (2018). Open source low-cost power monitoring system. HardwareX.

[32] Quintana, M., Lange, H. & Bergés, M. Design and implementation of a low-cost arduino-based high-frequency ac waveform meter board for the raspberry pi. *Proceedings of the 4th acm international conference on systems for energy-efficient built environments*, 34 (2017).

